# Clinical outcomes of ischemic and non-ischemic cardiomyopathy following catheter ablation for ventricular tachycardia among elderly patients

**DOI:** 10.1007/s10840-026-02316-8

**Published:** 2026-04-01

**Authors:** Kenji Hashimoto, Samual Turnbull, Max Bickley, Kaimin Huang, Kasun De Silva, Ashwin Bhaskaran, Saurabh Kumar

**Affiliations:** 1 Department of Cardiology, Westmead Hospital, Westmead Applied Research Centre, University of Sydney, Sydney, NSW 2145 Australia; 2 Cardiology Department, Westmead Hospital, Corner Hawkesbury and Darcy Roads, Westmead, Sydney, NSW 2145 Australia

**Keywords:** Cardiomyopathy, Ventricular tachycardia, Catheter ablation, Elderly patients

## Abstract

**Background:**

Catheter ablation (CA) is an effective treatment for ventricular tachycardia (VT), but its role in elderly non-ischemic cardiomyopathy (NICM) patients remains unclear. We aim to evaluate the feasibility of VT ablation in this population by comparing outcomes with elderly ischemic cardiomyopathy (ICM) patients.

**Methods:**

We retrospectively analyzed patients ≥ 70 years with structural heart disease who underwent VT ablation at a tertiary center from 2016 to 2024. Patients were categorized as NICM (*n* = 52) or ICM (*n* = 70), and demographic, mapping, procedural, and long-term outcomes were compared.

**Results:**

The mean age was 76.5 ± 5.2 and 76.6 ± 4.6 years in the NICM and ICM groups. The baseline demographic data were not different except for a lower left ventricular (LV) ejection fraction in the ICM group. The LV substrate map showed larger overall scars in ICM, whereas NICM had a relatively larger unipolar than bipolar scar, suggesting intramural substrate. In NICM, scars commonly involved the perivalvular region, with frequent basal septal involvement. During a median follow-up of 819 days, the rates of VA recurrence after multiple procedures and cardiovascular mortality were similar between the groups. The estimated one-year VA-free survival was 64.6 ± 7.0% in NICM and 74.9 ± 5.5% in ICM (*log-rank P* = 0.19), while cardiovascular mortality-free survival was 84.0 ± 5.2% and 85.7 ± 4.2%, respectively (*log-rank P* = 0.32). Furthermore, VA recurrence was associated with a higher risk of subsequent cardiovascular mortality (hazard ratio 2.12, 95% confidence interval 1.06–4.22, *P* = 0.03).

**Conclusion:**

CA for VT in elderly patients demonstrated comparable feasibility in both ICM and NICM. Effective VT control may contribute to favorable survival outcomes.

**Graphical Abstract:**

**Figure:**
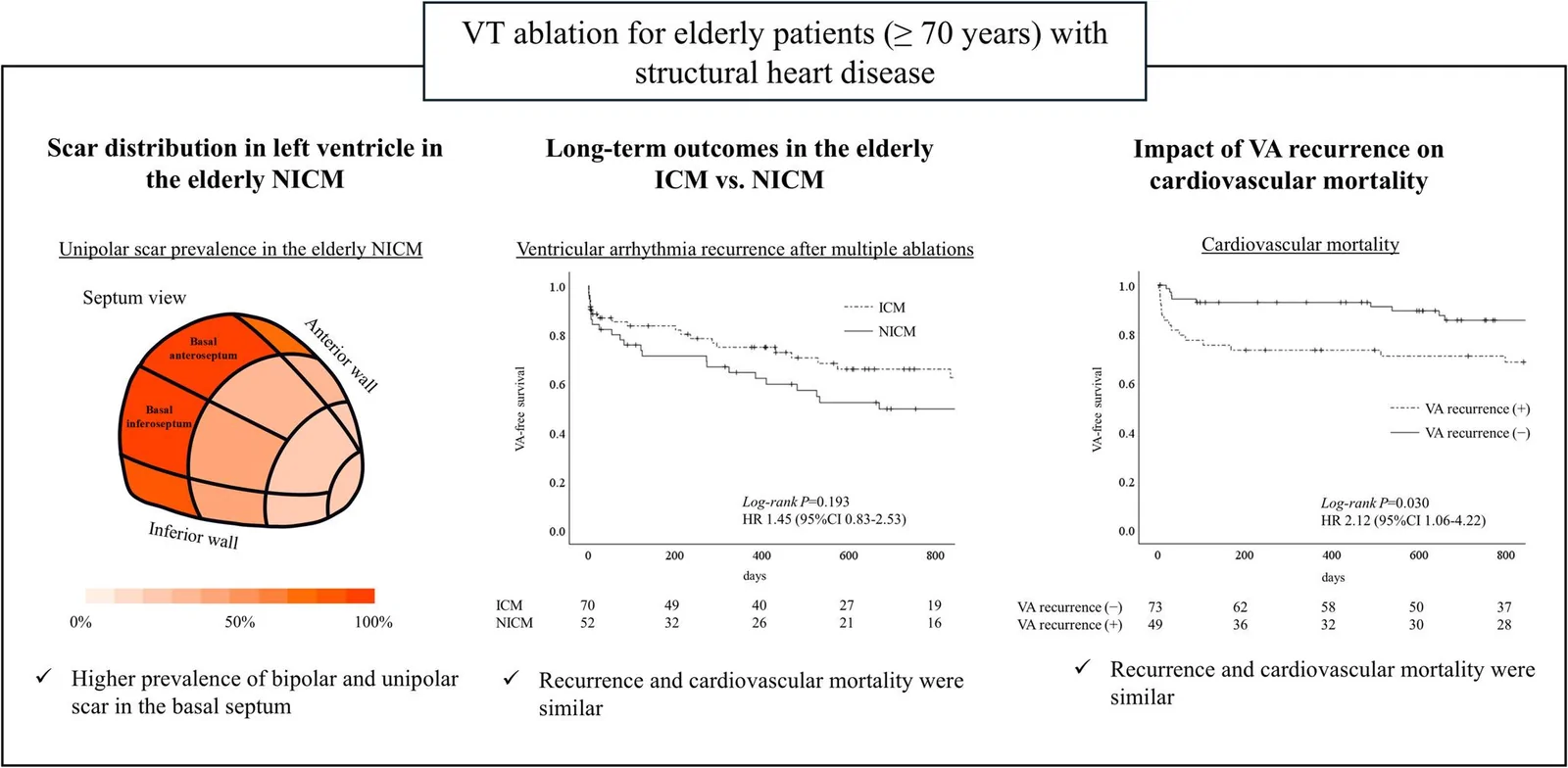
Abbreviation: CI, confidence intervals; HR, hazard ratio; ICM, ischemic cardiomyopathy; NICM, non-ischemic cardiomyopathy; VA, ventricular arrhythmia.

**Supplementary Information:**

The online version contains supplementary material available at https://doi.org/10.1007/s10840-026-02316-8.

## Introduction

Ventricular tachycardia (VT) is a major cause of death in patients with structural heart disease (SHD). Implantable cardioverter defibrillators (ICDs) reduce sudden cardiac death and are now widely used in SHD patients [1]. However, although ICDs reduce fatal outcomes by terminating VT episodes with anti-tachycardia pacing and shocks, they do not prevent the occurrence of VT itself. ICD shocks are not only painful but are also associated with worse survival outcomes [2]. Interventions to reduce the incidence of VT and ICD shocks are crucial for patients with recurrent VT. Catheter ablation (CA) has emerged as an effective therapy to reduce VT burden and appropriate ICD interventions [3]. 

The widespread use of ICDs in patients with SHD has led to longer survival rates, and consequently, the number of elderly patients with SHD and ICDs is expected to rise. Among these patients, some may experience recurrent VT and ICD shocks and may benefit from CA. However, CA for VT is an invasive procedure and is often avoided in the elderly population, who tend to have a relatively higher risk of non-sudden death and carry more significant comorbidities. A previous meta-analysis comparing the outcomes of VT ablation for elderly and young SHD patients showed that procedural-related complications and VA recurrence after CA were comparable; at the same time, the risk of all-cause mortality was higher in the elderly [4]. 

However, there is a notable paucity of studies specifically focusing on elderly patients with NICM. NICM is a diverse group of myocardial disorders encompassing a variety of etiologies, each presenting unique challenges in management, with outcomes that are also highly variable [5]. Despite its clinical significance, NICM remains less extensively studied than ischemic cardiomyopathy (ICM), and there is a paucity of data on the safety and efficacy of VT ablation in elderly patients with NICM.

In this study, we retrospectively analyzed consecutive SHD patients aged 70 years or older who underwent CA for VT. The objectives of this study are: (1) to describe the baseline characteristics, electroanatomic mapping (EAM) features, and ablation data in elderly NICM patients compared with elderly ICM patients, and (2) to compare the long-term outcomes between the two groups.

## Methods

### Patient cohort

Patients who presented with VT and underwent CA at a tertiary referral center (Westmead Hospital, Sydney) from January 2016 to July 2024 were retrospectively reviewed, and those with SHD aged ≥ 70 years were included in this study. NICM patients were identified as an absence of relevant coronary artery disease and defined according to the European Society of Cardiology Working Group on Myocardial and Pericardial Diseases [6]. ICM was defined as left ventricle (LV) dysfunction accompanied by significant coronary artery disease, characterized by at least one of the following: history of revascularization or acute myocardial infarction [7]. Patients with mixed cardiomyopathy, defined as patients in whom scar distribution had a non-coronary distribution, in a pattern consistent with NICM (e.g. isolated perivalvular scar, septal scar, epicardial lateral scar), with co-existent coronary artery disease, were excluded from this analysis [8]. The patient’s age at the time of the first CA procedure was used as the defining age. Demographic, procedural, and follow-up data were retrospectively collected from electronic medical records. The study was approved by the Western Sydney Local Health District Human Research Ethics Committee.

## Mapping and ablation

All procedures were performed under general anesthesia. Anti-arrhythmic drugs were stopped five half-lives before ablation, except for amiodarone and in urgent cases. Intravenous heparin (100 units/kg) was given as a bolus after achieving vascular access, with additional ad hoc boluses administered to maintain an activated clotting time > 350s. Non-invasive program stimulations were performed when the electrocardiogram morphology of targeting VTs was unclear before the procedure. A quadripolar catheter was deployed in the right ventricular (RV) apex, and an intracardiac echocardiography (ICE) catheter was inserted through the femoral vein. An SL3 sheath (Abbott Medical, Chicago, Illinois, USA) was used to insert a decapolar catheter into the coronary sinus. The endocardial LV was accessed either transeptally (Large curve Agilis, Abbott), or retrogradely (SL1 8.5Fr, Abbott) or both, by the operator’s discretion. The endocardial surface was mapped using a multi-electrode catheter (DecaNav or Pentarray, Biosense Webster, Diamond Bar, California, USA; or HD grid or Livewire, Abbott) with fluoroscopy and a three-dimensional EAM system (CARTO, Biosense Webster, Diamond Bar, California, USA; Ensite Precision or EnsiteX, Abbott) to guide catheter manipulation. In the EAM system and the CardioLab EP Recording System (GE Healthcare, Chicago, Illinois, USA), bandpass filtering was performed at 30–500 Hz for bipolar signals and 2–240 Hz for unipolar signals. An endocardial three-dimensional shell of chamber geometry was constructed for each ventricle with electrogram recordings during the sinus or paced rhythm. The CARTOSOUND module (SoundStar; Biosense Webster) was used in patients using CARTO system, and an anatomic three-dimensional reconstruction of the RV and left ventricle (LV) was created from sequential two-dimensional ICE slices acquired from the right atrium and ventricle. Epicardial access was considered in cases that had previously failed endocardial CA due to the absence of identifiable target sites and/or underwent pre-procedural imaging such as cardiac computed tomography and/or magnetic resonance imaging suggestive of epicardial VT scar.

To induce VT, programmed electrical stimulation was performed from the RV apex, using a 400 ms drive train with up to 4 extra-stimuli beginning at 300 ms and decrementing by 10 ms down to ventricular refractoriness. Clinical VT was defined by comparing induced VT with the 12-lead electrocardiograms of spontaneous VT recorded prior to the procedure. When 12-lead ECGs of spontaneous VT were unavailable, stored ICD electrograms were referred for comparison instead. When VTs were not hemodynamically tolerated, ablation targets were identified using pace mapping, as well as mapping of late, isolated, or fractionated potentials, and/or late abnormal ventricular activities [9]. Ablation was performed using an irrigated radiofrequency catheter (ThermoCool SmartTouch Surround Flow, Biosense Webster; TactiCath SE or TactiFlex SE, Abbott) at standard power settings of 30–50 Watts, aiming for an impedance fall of 10–20 ohms at each lesion. Acute procedural success was evaluated with programmed extra stimulation from the RV with up to 4 extra-stimuli down to ventricular refractoriness or 200 msec, with or without isoprenaline at the end of the ablation, and non-inducibility of VTs was assessed. The first ablation procedure in each patient at our institution during the study period was considered as the index procedure for this study and was assessed.

## Scar assessment

The EAM maps during sinus or paced rhythm were assessed to elucidate scar characteristics; Partial substrate maps, which did not cover the entire chamber surface, were excluded from the assessment. The chamber mapped was based on the induced or spontaneously occurring VT characteristics and findings on pre-procedural imaging. Bipolar and unipolar voltage was defined as the peak-to-peak electrogram voltage. The voltage maps were evaluated based on criteria previously reported: < 0.5mV for dense scar and < 1.5mV for low voltage zone (LVZ) in bipolar endocardial map, and < 5.5mV and < 8.3mV for LVZ in RV and LV unipolar endocardial map, respectively [10–12]. The LVZ area was calculated utilizing the EAM system. The ratio between bipolar and unipolar LVZ was calculated to assess the transmurality of the scar, and the ratio between dense scar and LVZ was calculated to assess the heterogeneity of the scar. In the NICM group, the distribution of the LVZ was visually assessed, using an 8-segment model in the RV and a 17-segment model in the LV [13, 14]. 

## Outcomes

The start of follow-up was the date of the first VT ablation procedure at our institution. Patients were followed until death or loss of follow-up. Follow-up included a review of records of hospital and outpatient clinic visits and home monitoring data. The primary efficacy outcomes were cardiovascular mortality. The secondary efficacy outcome was ventricular arrhythmia (VA) recurrence after single and multiple ablations. The recurrence of VA was defined as VT lasting more than 30 s or sustained VT or ventricular fibrillation requiring ICD therapies. The primary safety outcome was procedural-related complications, defined as any adverse event requiring escalation of care or increased length of inpatient stay.

### Statistical analysis

The Statistical Package for the Social Sciences version 29 (IBM Corp., Armonk, NY) was used for analysis. Continuous variables were expressed as mean ± standard deviation if normally distributed; median and 25–75% interquartile range (IQR) were used if the data were not normally distributed. Categorical variables were expressed as frequencies and percentages. Continuous variables were compared using a t-test when normally distributed or a Mann–Whitney U test when not normally distributed. A chi-squared test was used when comparing categorical variables or Fisher’s exact test when required. The estimated event-free survival probabilities were calculated using a Kaplan–Meier method, and log-rank tests were used for group comparisons. Cox proportional hazard models were created to calculate hazard ratios (HR) and 95% confidence intervals (CI) for the efficacy outcomes. To examine the impacts of recurrent VA on subsequent clinical outcomes, cardiovascular mortality was compared between patients who experienced VA recurrence and those who did not. A two-tailed P-value < 0.05 was considered statistically significant.

## Results

### Study participants

Among 482 VT ablation procedures in 332 patients with SHD from January 2016 to July 2024, the present analysis included 122 patients aged 70 years or older; 52 patients in the NICM group and 70 patients in the ICM group, after excluding ten patients with mixed cardiomyopathy. Patient characteristics are shown in Table 1. The mean ages were 76.5 ± 5.2 years old in the NICM group and 76.6 ± 4.6 years old in the ICM group (*P* = 0.93). The ICM group had a lower LV ejection fraction (33.3 ± 12.4% vs. 42.7 ± 13.6%, *P* < 0.01); other baseline characteristics, including comorbidities, were not significantly different between the two groups. The NICM group comprised 42 (81%) idiopathic NICM, four (8%) valvular cardiomyopathy related to valvular heart disease, two (4%) hypertrophic cardiomyopathy, one (2%) cardiac sarcoidosis, one (2%) cardiac amyloidosis, one (2%) congenital cardiomyopathy, and one (2%) chemotherapy-related cardiomyopathy.

**Table 1:** Baseline characteristics

	ICM *n* = 70	NICM *n* = 52	*P* value
Age, years	76.6 (4.6)	76.5 (5.2)	0.925
Male, *n*, %	65 (92.9)	44 (84.6)	0.234
BMI, kg/m^2^	28.1 (4.6)	29.4 (6.1)	0.218
LVEF, %	33.3 (12.4)	42.7 (13.6)	< 0.001
Electrical storm, *n*, %	46 (65.7)	29 (55.8)	0.347
Antiarrhythmic drugs, *n*, %	52 (74.3)	38 (73.1)	1
Amiodarone, *n*, %	36 (51.4)	26 (50.0)	1
Sotalol, *n*, %	14 (20.0)	10 (19.2)	1
Mexiletine, *n*, %	11 (15.7)	6 (11.5)	0.603
Beta-blocker, *n*, %	56 (81.2)	42 (80.8)	1
Hemoglobin level, g/dl	13.5 (1.7)	13.5 (1.8)	0.885
Creatinine level, mg/dl	1.1 (0.4)	0.99 (0.3)	0.096
Diabetes, *n*, %	21 (30.4)	16 (30.8)	1
Hypertension, *n*, %	46 (66.7)	38 (73.1)	0.551
Hyperlipidemia, *n*, %	47 (68.1)	29 (55.8)	0.187
Chronic kidney disease, *n*, %	24 (34.3)	22 (42.3)	0.570
Atrial fibrillation, *n*, %	37 (53.6)	28 (53.8)	1
ICD, *n*, %	48 (68.4)	38 (73.1)	0.689
CRTD, *n*, %	12 (17.1)	6 (11.5)	0.448

Data are reported as mean (standard deviation) or number (%)

*Abbreviations:BMI* body mass index, *CRTD* cardiac resynchronization therapy defibrillator, *ICD* implantable cardioverter defibrillator, *LVEF* left ventricular ejection fraction, *SD* standard deviation, *SICD* subcutaneous implantable cardioverter defibrillator

## Scar assessment

**Fig. 1:**
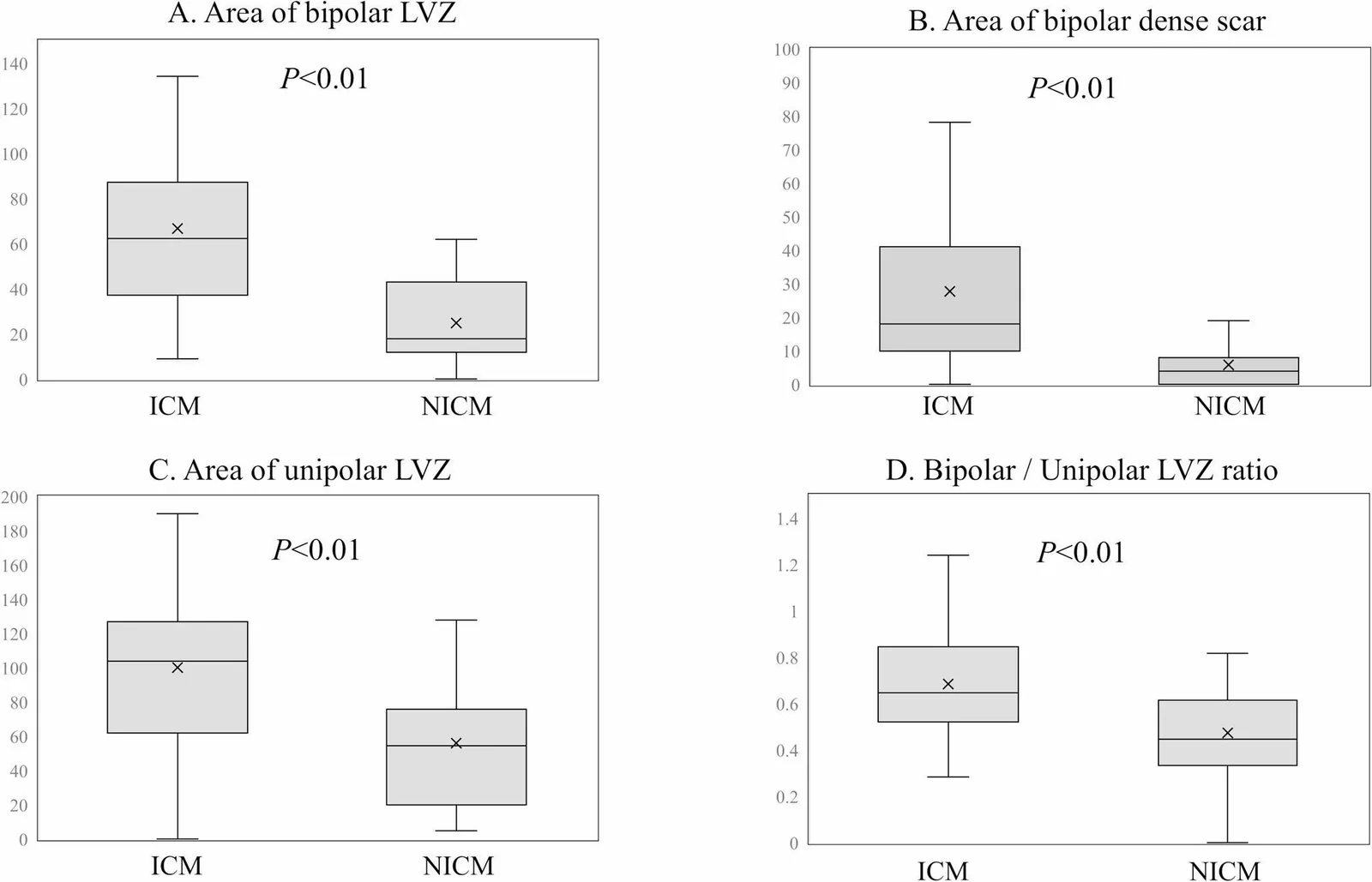
Comparison of scar area between ischemic and non-ischemic cardiomyopathy. **A** Bipolar low voltage zone (LVZ) was larger in the ICM group (median [interquartile range]: 63.2 [37.2, 87.0] vs 18.0 [12.0, 43.0] cm^2^, *P*<0.01). **B** Bipolar dense scar was larger in the ICM group (median [interquartile range]: 18.0 [10.0, 37.0] vs 4.0 [0, 8.0] cm^2^, *P*<0.01). **C** Unipolar LVZ was larger in the ICM group (median [interquartile range]: 104.0 [62.3, 126.8] vs 54.6 [20.0, 76.0] cm^2^, *P*<0.01). **D** The bipolar–unipolar LVZ ratio was lower in the NICM group (median [IQR]: 0.65 [0.52–0.84] vs. 0.45 [0.33–0.62], *P*<0.01), indicating that, relative to the bipolar LVZ, the unipolar LVZ was larger in the NICM group, despite its smaller absolute size. Abbreviations: ICM, ischemic cardiomyopathy; LVZ, low voltage zone; NICM, non-ischemic cardiomyopathy

**Fig. 2:**
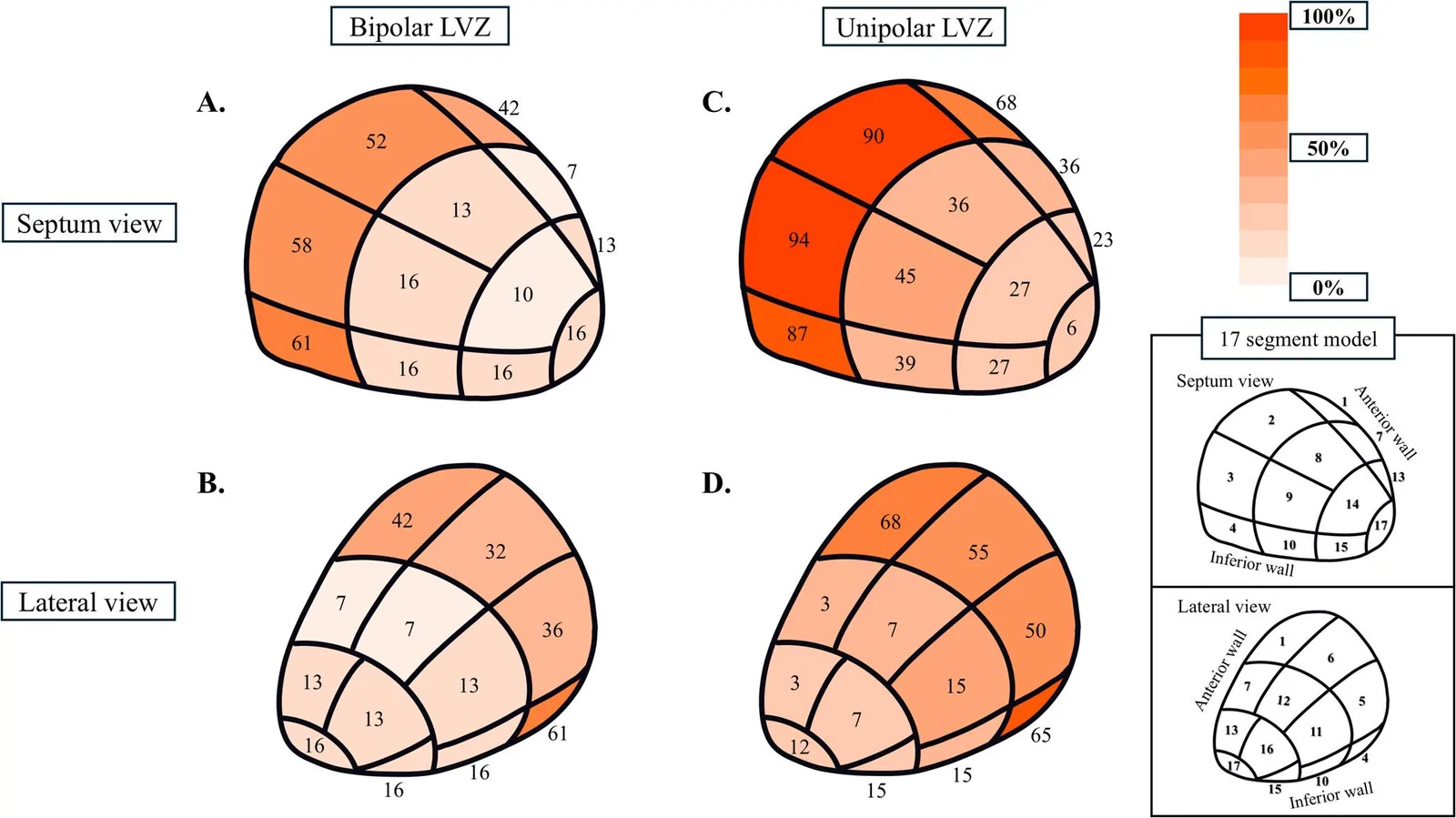
Distribution of low voltage zone in left ventricular bipolar and unipolar voltage map in non-ischemic cardiomyopathy. Among the patients with left ventricular scar, the percentages of low voltage zone (LVZ) in each segment are shown. The bright color indicates higher percentage as shown in a color bar. **A** Septum view in the bipolar voltage map. **B** Lateral view in the bipolar voltage map. **C** Septum view in the unipolar voltage map. **D** Lateral view in the unipolar voltage map. Perivalvular LVZ is commonly present, predominantly in the unipolar voltage map. Abbreviations: LVZ, low voltage zone

Substrate mapping of the entire LV was performed in 33 patients (64%) in the NICM group and 53 patients (76%) in the ICM group, allowing for the assessment of scar area and distribution. The LVZ identified in either bipolar or unipolar map was observed in 31 patients (94%) in the NICM group and 53 patients (100%) in the ICM group. Among patients with any LVZ in the LV, the area of scar in LV bipolar and unipolar maps was analyzed and compared between the two groups. The area of LVZ and dense scar was significantly larger in both bipolar and unipolar voltage maps in the ICM group (Fig. 1). The median (IQR) dense scar-LVZ ratio in the bipolar voltage map was lower in the NICM group (0.20 [0.07, 0.31] vs. 0.36 [0.22, 0.54], *P* < 0.01), indicating the proportion of borderline scar between 0.5mV and 1.5mV was higher in the NICM group. The median (IQR) bipolar-unipolar ratio was significantly lower in the NICM group, indicating the large unipolar scar in relation to the bipolar scar in the NICM group (0.65 [0.52, 0.84] vs. 0.45 [0.33, 0.62], *P* < 0.01). The median (IQR) number of segments involving bipolar and unipolar LVZ was higher in the ICM group (6 [5, 9] vs. 3 [1, 6], *P* < 0.01 in the bipolar map; 10 [6, 13] vs. 6 [4, 12], *P* = 0.038 in the unipolar map). The distribution of LVZ in bipolar and unipolar voltage maps was assessed in the NICM group. The LVZ was predominantly located in the perivalvular region; however, it was more extensive in the unipolar voltage map, compared to the bipolar voltage map (Fig. 2). In the NICM group, preprocedural cardiac magnetic resonance imaging performed within one year prior to the index procedure was available in four cases. (1) Idiopathic NICM patient: late gadolinium enhancement (LGE) was observed in the basal to mid inferoseptum with an intramural pattern at the basal level and a subepicardial pattern at the mid-level. The LV substrate map demonstrated small bipolar and unipolar scar areas in the basal inferoseptum and inferior wall. (2) Idiopathic NICM patient: LGE was present in the basal to mid inferoseptum with a subendocardial pattern, and in the anteroseptum with an intramural pattern. The LV substrate map showed bipolar and unipolar scar area in the basal anteroseptum and inferoseptum. (3) Idiopathic NICM patient: LGE was observed in the inferolateral wall, with an intramural pattern at the basal level and a transmural pattern at the apical level. The LV substrate map demonstrated normal bipolar and unipolar LV voltages; however, ICE revealed regional echobrightness corresponding to this area. (4) Cardiac sarcoidosis patient: Patchy LGE involved the basal to mid anteroseptum, as well as the basal anterolateral, inferolateral, and inferoseptal regions. The LV substrate map revealed an extensive basal scar, with the unipolar abnormality being more widespread than the bipolar abnormality.

**Fig. 3:**
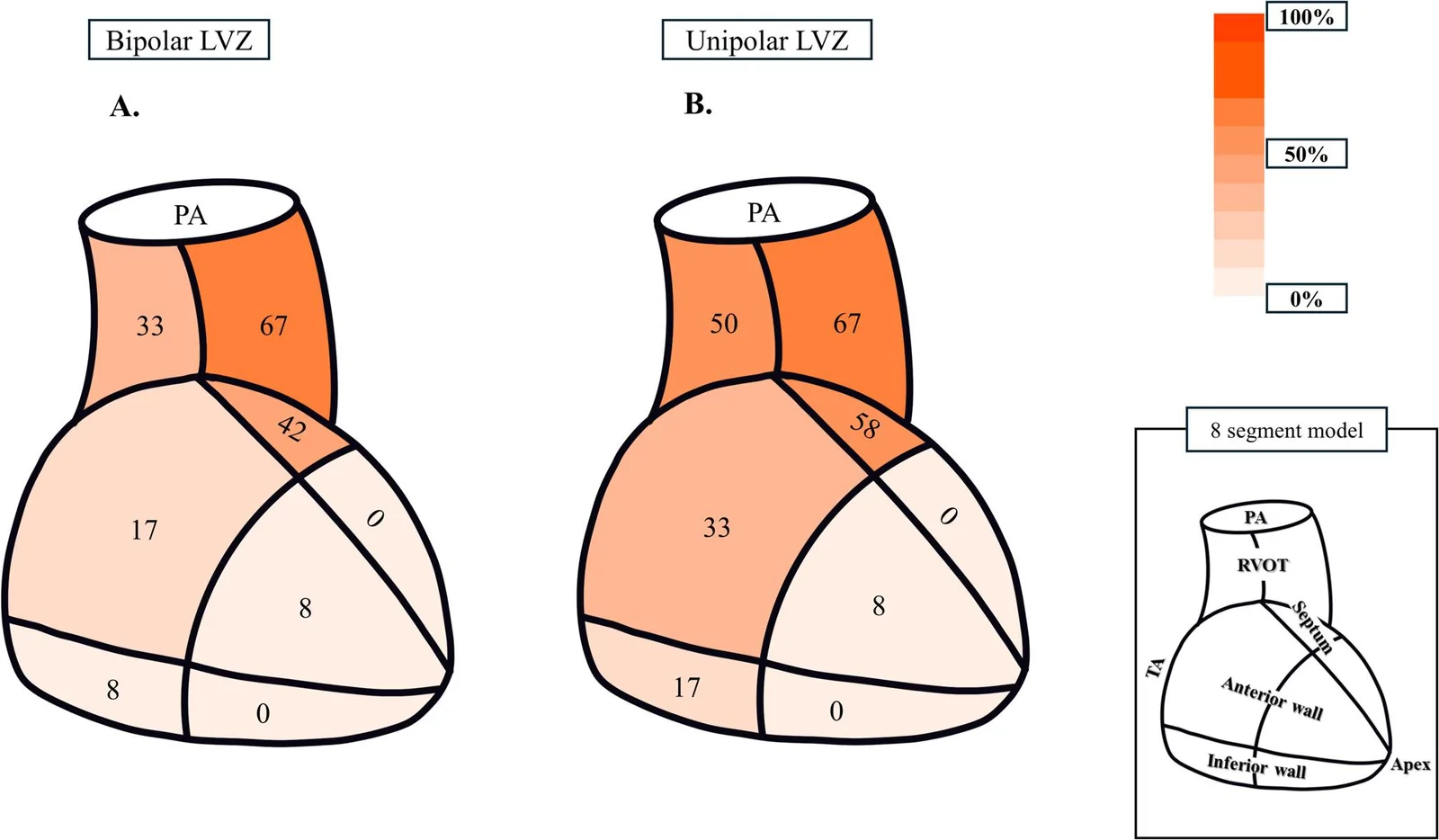
Distribution of low voltage zone in right ventricular bipolar and unipolar voltage map in non-ischemic cardiomyopathy. Among the patients with right ventricular scar, the percentages of low voltage zone in each segment are shown. The bright color indicates higher percentage as shown in a color bar. **A** Bipolar low voltage zone. **B** Unipolar low voltage zone. LVZ predominantly present at septum and more extensive in unipolar voltage map. Abbreviations: LVZ, low voltage zone; PA, pulmonary annulus; RVOT, right ventricular outflow tract; TA, tricuspid annulus

Substrate mapping of the entire RV was performed in 15 patients (28%) in the NICM group, allowing for the assessment of scar area and distribution. The LVZ identified in either the bipolar or unipolar map was observed in 12 patients (75.0%). Among patients with any LVZ in the RV, the distribution of LVZ in RV bipolar and unipolar maps was assessed. The median (IQR) bipolar LVZ, bipolar dense scar, and unipolar LVZ were 13.3 (8.9, 23.5) cm^2^, 3.0 (1.5, 9.3) cm^2^, and 24.8 (15.3, 33.0) cm^2^, respectively. The median (IQR) bipolar-unipolar LVZ ratio was 0.55 (0.33, 0.99). Distributions of bipolar and unipolar LVZ in the RV were shown in Fig. 3. LVZ was predominantly present at the basal and RV outflow tract septum, and it was more extensive in the unipolar voltage map, compared to the bipolar voltage map. The median (IQR) numbers of segments involving LVZ were 2 (1, 2) in the bipolar voltage map, and 2 (2, 3) in the unipolar voltage map (*P* = 0.02).

### Ablation procedure

In the NICM group, 42 (81%) patients underwent a single VT ablation, six (12%) underwent two procedures, and four (8%) underwent three procedures. In the ICM group, 54 (77%) patients underwent a single VT ablation, 12 (17%) underwent two (3%) procedures, two (3%) underwent three procedures, and two (3%) underwent four procedures during the study period. During the study period, one (2%) in the NICM group, and three (4%) in the ICM group underwent epicardial mapping and ablation (*P* = 0.46). Half normal saline irrigation was used in 6 patients (12%) of the NICM patients and 14 patients (20%) of the ICM patients in the index procedures (*P* = 0.23), and in 7 patients (14%) of the NICM and 18 patients (26%) of the ICM when including repeated procedures (*P* = 0.12). One ICM patient underwent a surgical cryoablation due to the intramural VT circuit. Bipolar ablation or ethanol infusion was not performed in our study cohort. The first ablation procedure in each patient at our institution in the study period was considered the index procedure for this study and was assessed, which is shown in Table 2.

**Table 2:** Procedural data

	ICM	NICM	*P* value
VT induced, *n*, %	61 (91.0)	42 (84.0)	0.265
Number of VTs, *n*, (mean ± SD)	3.1 (1.9)	4.2 (3.6)	0.067
Average TCL, ms, (mean ± SD)	353 (83)	349 (71)	0.801
Minimum TCL, ms, (mean ± SD)	312 (73)	301 (53)	0.353
Maximum TCL, ms, (mean ± SD)	395 (109)	406 (116)	0.624
PES at the end, *n*, %	52 (76.5)	40 (80.0)	0.823
Non-inducibile VT, *n*, %	34 (65.0)	22 (55.0)	0.525
RF duration, sec, (mean ± SD)	3124 (1355)	2479 (1609)	0.026
Procedure duration, min, (mean ± SD)	249 (96)	203 (64)	0.003
Fluoroscopy time, sec, (mean ± SD)	947 (649)	766 (362)	0.103
ECMO use, *n*, %	2 (2.9)	0 (0)	0.507
Atrioventricular block, *n*, %	4 (5.7)	7 (13.5)	0.202
Complication, *n*, %	10 (14.3)	5 (9.6)	0.580
In-hospital mortality, *n*, %	7 (10.0)	3 (5.8)	0.514

Data are reported as mean (standard deviation), or number (%)

*Abbreviations:* *ECMO* extracorporeal membrane oxygenation, programmed electrical stimulation, *RF* radiofrequency, *SD* standard deviation, *TCL* tachycardia cycle length, *VT* ventricular tachycardia

More VTs were induced in the NICM group, though not significantly (4.2 ± 3.6 vs. 3.1 ± 1.9, *P* = 0.067). The average, maximum, and minimum tachycardia cycle length were comparable between the two groups. At the end of procedures, programmed electrical stimulation was performed in 80% of the NICM group and 77% of the ICM group, which exhibited comparable non-inducibility of any VT (55% vs. 65%, *P* = 0.53) and clinical VT (90% vs. 95%, *P* = 0.39). The reasons for not performing programmed electrical stimulation at the end of the procedure were non-inducible VT at baseline in 19 patients and operator discretion due to patient condition (e.g., hemodynamic instability) in 11 patients. Among patients who underwent programmed electrical stimulation with up to 4 extra-stimuli, those with inducible VT at the end of the procedure had a significantly higher number of inducible VTs than those without inducible VT (4.7 ± 3.8 vs. 2.8 ± 1.7, *P* = 0.035). On the other hand, age, LV ejection fraction, presence of electrical storm, and bipolar and unipolar LV scar size in the substrate map were not significantly different. These results were consistent in the ICM and NICM subgroups.

Radiofrequency duration and total procedure duration were significantly longer in the ICM group (3124 ± 1355 vs. 2479 ± 1609 s, *P* = 0.026; 249 ± 96 vs. 203 ± 64 min, *P* < 0.01). Ablation at the basal septal substrate near the conduction system was required and resulted in anticipated atrioventricular block in seven patients (14%) in the NICM group and four patients (6%) in the ICM group (*P* = 0.20). Extracorporeal membrane oxygenation was utilized in two ICM patients (3%) but in no NICM patient (*P* = 0.51). The procedural-related complication rate was not significantly different between the NICM and the ICM group (10% vs. 14%, *P* = 0.58), as summarized in Supplemental Table S1. Three (6%) in-hospital mortalities occurred in the NICM group, while seven (10%) in the ICM group (*P* = 0.51).

### Clinical Outcomes

The median (IQR) days to discharge after the procedures were 5 (2, 7) days in the NICM group, and 3 (1, 6) days in the ICM group (*P* = 0.24). At discharge, anti-arrhythmic drugs were prescribed in 53% of the NICM group and 51% of the ICM group, while amiodarone was used in 41% and 38%, respectively (both *P* = 0.85). At one year after the last procedure, anti-arrhythmic drugs were used in 40% of the NICM group and 43% of the ICM group (*P* = 0.84), while amiodarone was used in 25% and 32%, respectively (*P* = 0.50). Among patients who were free of VA recurrence after the last procedure, anti-arrhythmic drugs were used in 42% of the NICM group and 34% of the ICM group (*P* = 0.79), while amiodarone was used in 24% and 21%, respectively (*P* = 0.55).

The median (IQR) follow-up period was 838.5 (155.5, 1351) days in the NICM group, and 786 (382, 1476) days in the ICM group after the index ablation procedure (*P* = 0.73). VA recurred after a single procedure in 27 (52%) NICM patients and 36 (51%) ICM patients, while VA recurred after multiple procedures in 25 (48%) NICM patients and 24 (34%) ICM patients. Among 25 NICM patients who experienced VA recurrence after multiple procedures, 21 had idiopathic NICM, while one each had cardiac sarcoidosis, cardiac amyloidosis, congenital heart disease, and chemotherapy-induced cardiomyopathy. In the NICM patients with LV substrate maps, scar distribution was compared between those with and without VA recurrence (*n* = 16 and *n* = 17, respectively). Bipolar scar in segment 1 (basal anterior wall), and unipolar scar in segment 2 (basal anteroseptum) were more prevalent in patients with VA recurrence than in those without VA recurrence (63% vs. 18%, *P* = 0.013; 71% vs. 100%, *P* = 0.044, respectively). On the other hand, in the RV substrate map, no significant difference was identified.

**Fig. 4:**
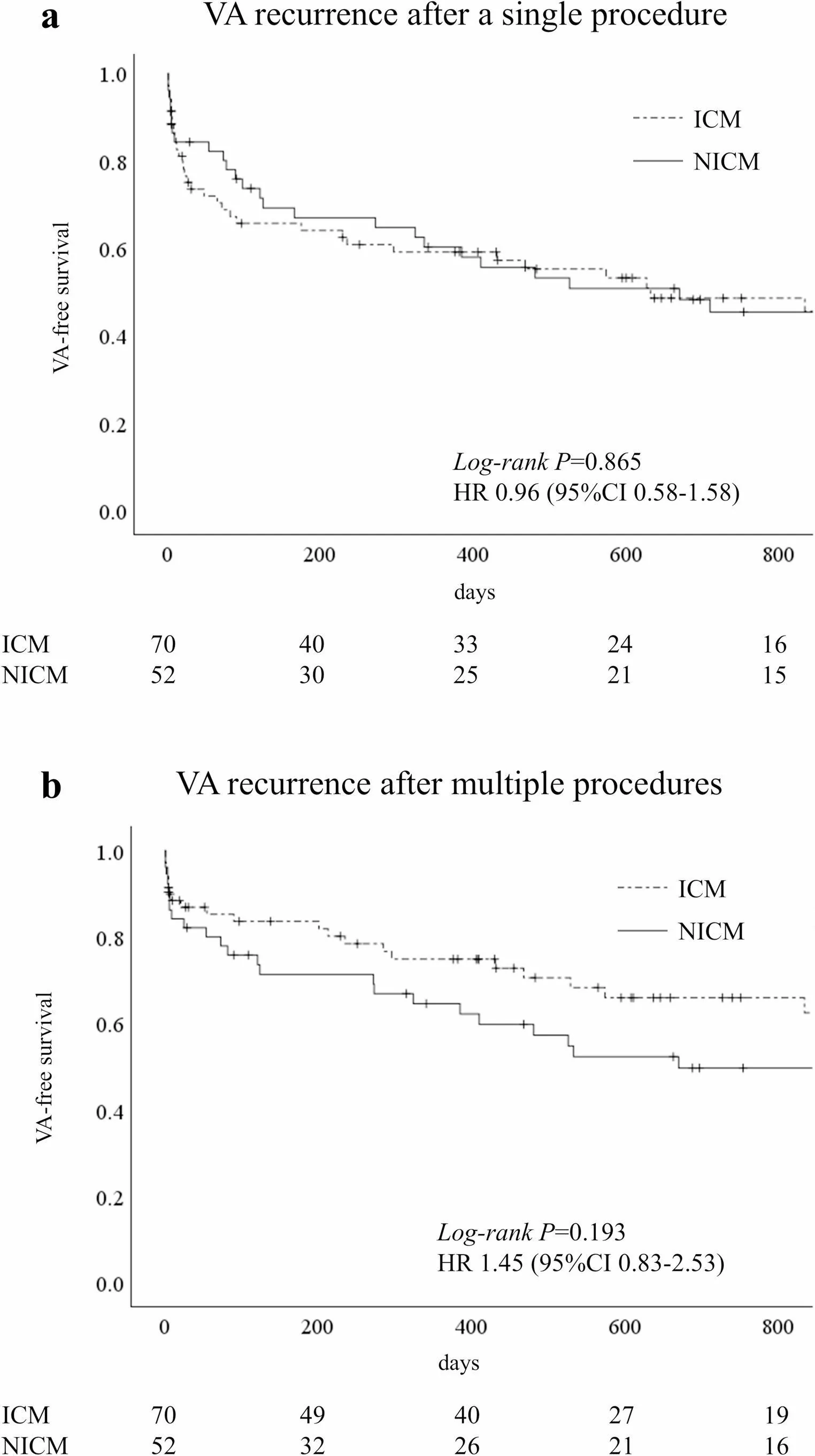
Event-free survival analysis for ventricular arrhythmia recurrence. The cumulative incidence of ventricular arrhythmia (VA) recurrence after single and multiple procedures was comparable between the two groups (panel **a** and **b** respectively). Abbreviations: CI, Confidence interval; HR, hazard ratio; VA, ventricular arrhythmia

**Fig. 5:**
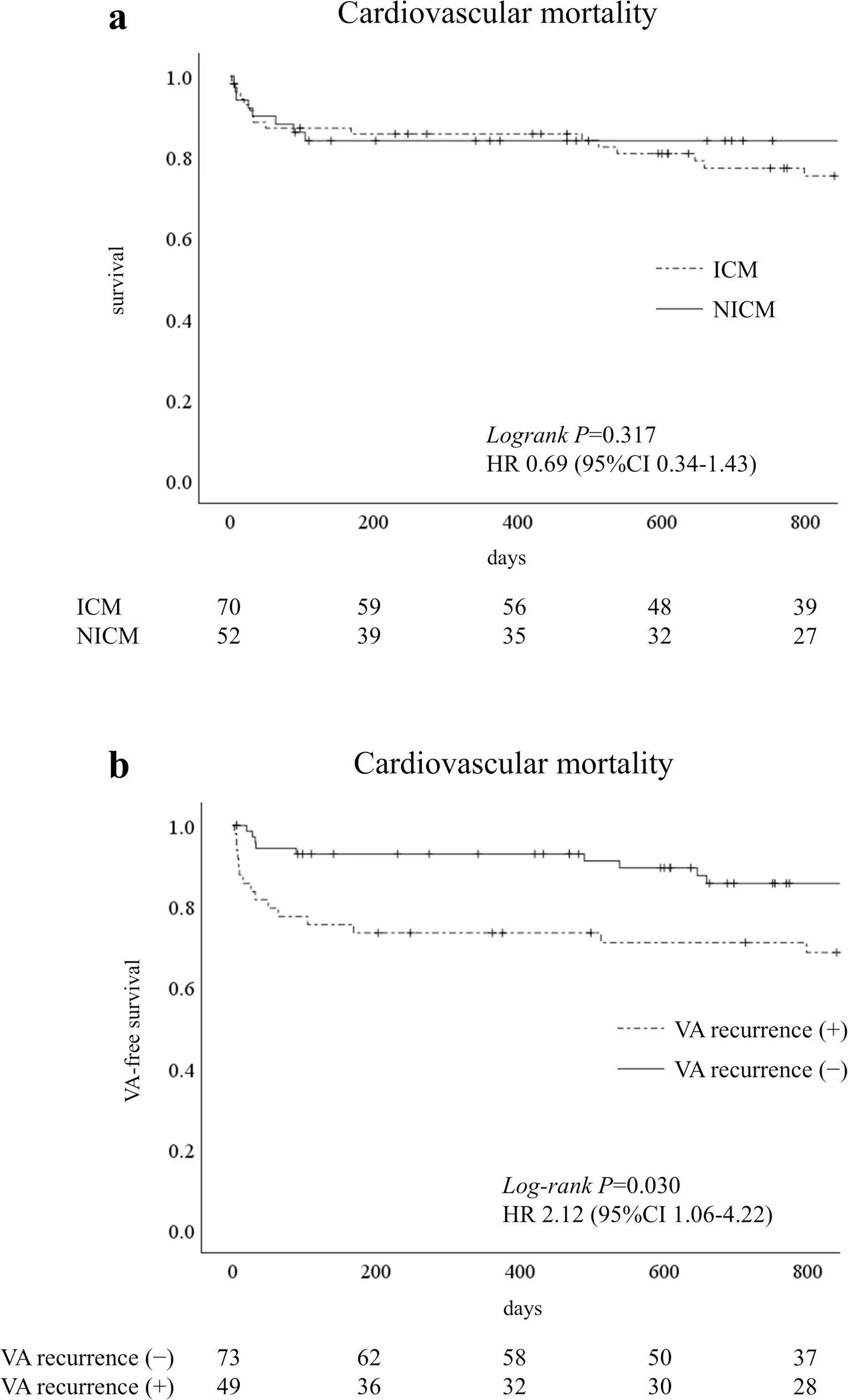
Event-free survival analysis for cardiovascular mortality. **a**. The cumulative incidence of the composite of cardiovascular mortality after a procedure was comparable between the two groups. **b**. VA recurrence after procedures was associated with subsequent cardiovascular mortality. Abbreviations: CI, Confidence interval; HR, hazard ratio; VA, ventricular arrhythmia

Event-free survival analysis indicated comparable VA recurrence rates after single and multiple procedures between the NICM and ICM groups (*log-rank P* = 0.87, HR 0.96, 95% CI 0.58–1.58; and *log-rank P* = 0.19, HR 1.45, 95% CI 0.83–2.53, respectively). The estimated one-year VA-free survival rates after single and multiple procedures were 60.3 ± 7.1% and 64.6 ± 7.0% in the NICM group, and 59.2 ± 6.1% and 74.9 ± 5.5% in the ICM, respectively (Fig. 4). Cardiovascular mortality occurred in 11 (21%) in the NICM group, and 23 patients (33%) in the ICM group. Event-free survival analysis indicated comparable cardiovascular mortality rates between the NICM and ICM groups (*log-rank P* = 0.32, HR 0.69, 95% CI 0.34–1.43). The estimated one-year cardiovascular mortality-free survival rates were 84.0 ± 5.2% in the NICM group and 85.7 ± 4.2% in the ICM group (Fig. 5). In addition, the risk of cardiovascular mortality was compared between the patients who had experienced VA recurrence after multiple procedures and those who had not. Event-free survival analysis indicated VA recurrence was associated with significantly higher cardiovascular mortality risk (*log-rank P* = 0.03, HR 2.12, 95% CI 1.06–4.22). The estimated one-year cardiovascular mortality-free survival rates were 73.5 ± 6.3% in the patients with VA recurrence, and 93.0 ± 3.0% in those without VA recurrence (Fig. 5).

## Discussion

This retrospective observational study included consecutive SHD patients aged 70 years or older who underwent CA for VT, and compared the efficacy and safety outcomes between ICM and NICM. The main findings are as follows:

1. VT ablation was feasible in elderly patients with both ICM and NICM, with acceptable efficacy and safety outcomes.
2. In this elderly cohort, VA recurrence after multiple procedures was associated with higher cardiovascular mortality, highlighting the importance of effective VA control.
3. Scar characteristics on voltage mapping differed significantly between the two groups. The ICM group exhibited larger overall scar areas, whereas the NICM group had smaller scar areas but a smaller bipolar-to-unipolar LVZ ratio, suggesting a predominance of intramural or epicardial scar in this population.

Previously, Inada et al. investigated the clinical outcomes of VT ablation in elderly patients aged 75 years and over with coronary artery disease and indicated that the VA recurrence, mortality, and procedural-related complications were comparable to those of patients younger than 75 years [15]. Liang et al. categorized SHD patients who underwent CA into three groups: younger than 65 years, from 65 to 75 years, and older than 75 years. They reported that acute procedural success, complications, 28-day survival, and one-year VT-free survival were similar in the three groups. However, the older groups had worse survival at long-term follow-up [16]. Frontera et al. presented outcomes after VT ablation among elderly SHD patients (≥ 80 years), where octogenarians had more procedural-related complications and more long-term VT recurrence, even though one-year VT-free survival was similar to younger patients (< 80 years) [17]. Vakil et al. analyzed a large international multi-center VT ablation registry and reported that older SHD patients (≥ 70 years) had a higher all-cause mortality rate but a comparable VA recurrence rate compared to the younger patients (< 70 years). They also performed a sensitivity analysis stratified by 5-year age increments in patients ≥ 70 years, which showed similar VT recurrence-free survival following VT ablation [18]. Overall, one-year VT-free survivals after VT ablation in these studies have been reported at approximately 70%. Our results (one-year VA-free survival rate of 75% in the ICM group and 65% in the NICM group) were consistent with these prior reports, reinforcing the efficacy of VT ablation in the elderly cohort. However, data on NICM patients remain limited, particularly regarding demographics, substrate distribution, and procedural outcomes, as most prior studies included NICM but did not analyze them separately from ICM. In contrast, our study directly compared 52 consecutive NICM patients with 70 ICM patients. In the general population, including young patients, NICM was reported to be associated with a higher VT recurrence rate and a lower mortality rate, compared to ICM [19, 20]. On the contrary, our findings demonstrated that the risks of VA recurrence and cardiovascular mortality were comparable between the ICM and NICM groups, suggesting the efficacy of VT ablation in NICM patients as well as ICM patients in the elderly cohort. The safety outcomes, including procedure-related complications, were similar in the two groups, with 14% in the ICM and 10% in the NICM. However, it is noteworthy that seven (10%) in-hospital mortalities occurred in the ICM group and three (6%) in the NICM group, suggesting the high-risk profile of this elderly cohort. It highlights the need for careful patient selection and management in elderly patients undergoing VT ablation. Furthermore, since we did not have a comparison arm with medically treated patients, the efficacy and safety of CA compared to medical therapy were unknown. However, over 70% of the patients had anti-arrhythmic drugs at the time of the procedure. Our results suggest that CA provides an important treatment strategy along with medical therapy for elderly patients with recurrent VT. We also assessed patients who experienced VA recurrence after multiple procedures, indicating the higher risk of cardiovascular mortality in these patients, which was consistent with previous data [18]. Our findings strengthen the evidence that successful ablation leads to favorable survival outcomes in the elderly population.

Of note, we also analyzed scar characteristics on EAM in both groups. In the LV substrate map, overall scar areas, including bipolar LVZ, bipolar dense scar and unipolar LVZ, were significantly larger in the ICM group compared to those in the NICM group. On the other hand, the NICM group exhibited overall smaller scar areas, but unipolar LVZ in relation to bipolar LVZ was larger, indicating epicardial and intramural scar. These findings are consistent with a previous study, investigating the substrate difference between ICM and NICM regardless of age [20]. Furthermore, in the NICM group, the distribution of LVZ in the bipolar and unipolar voltage maps was assessed (Fig. 2). The distribution of bipolar and unipolar scar was similar to the overall NICM population reported in the previous studies, predominantly involving the perivalvular region at the septum. In the RV substrate map, the scar involved the basal and RV outflow tract septum, which is consistent with an intramural septal substrate. Collectively, our findings indicate a high prevalence of basal septal involvement in elderly NICM patients, which may contribute to the limited procedural success of ablation due to complex intramural VT circuits [21, 22]. In fact, basal anteroseptal scars in the unipolar voltage map were associated with VA recurrence in our NICM cohort.

### Limitations

This analysis has several limitations. First, as this is a retrospective study, selection bias could impact the result. Physicians may be more likely to perform CA in elderly patients with relatively high functional status while avoiding the procedure in those with frailty.　However, frailty was not systematically assessed due to insufficient data to calculate a formal frailty index. Second, the data were derived from a single tertiary referral centre, and the treatment approach was left to the discretion of the treating physician, which may limit its generalizability. Particularly, the procedures were routinely performed under general anaesthesia. The clinical outcomes may vary without general anaesthesia. Third, EAM analysis was performed in the patients who underwent substrate mapping covering the entire chamber rather than in all the included patients. The partial substrate map that did not cover the entire chamber surface was not included in the assessment. The decision to perform LV and/or RV substrate mapping was left to the operator’s decision. Furthermore, preprocedural cardiac magnetic resonance imaging was only available in four NICM patients; thus, the correlation between preprocedural imaging and EAM was not assessed. Accordingly, the results of our scar distribution might contain bias and should be interpreted cautiously. Fourth, programmed electrical stimulation was performed using a 400-ms drive train, and alternative drive trains or additional stimulation sites were not routinely employed, which may have contributed to failure to induce some VTs. Finally, no medically treated patients were included in our study, and approximately one-third of patients received amiodarone one year after the last procedure. Thus, whether catheter ablation is more effective or safer than medical treatment could not be assessed in our study. Instead, we focused on describing real-world clinical outcomes in ICM and NICM, as well as scar distribution in elderly NICM patients. Nevertheless, to determine whether catheter ablation should be preferred for elderly patients presenting with VT, further studies directly comparing catheter ablation and medical therapy are warranted.

## Conclusion

In our series of patients aged 70 years or older who underwent VT ablation, the risk of VA recurrence and procedural-related complications in the NICM was comparable to the ICM. Furthermore, our study indicated that VA recurrence following VT ablation was associated with a higher risk of subsequent cardiovascular mortality, underscoring the importance of VA control in this elderly cohort.

## Supplementary Information

Supplementary Material 1.

## Data Availability

Available from the corresponding author on reasonable request.

## Abbreviations

CA: Catheter Ablation
CI: Confidence Interval
EAM: Electroanatomic Mapping
HR: Hazard Ratio
ICD: Implantable Cardioverter Defibrillator
ICE: Intracardiac Echocardiography
ICM: Ischemic Cardiomyopathy
IQR: Interquartile Range
EAM: Electro-anatomical Mapping
LGE: Late Gadolinium Enhancement
LV: Left Ventricle
LVZ: Low Voltage Zone
NICM: Non-ischemic Cardiomyopathy
RV: Right Ventricle
SHD: Structural Heart Disease
VA: Ventricular Arrhythmia
VT: Ventricular Tachycardia
