# Supplementary material for: Clinical outcomes of ischemic and non-ischemic cardiomyopathy following catheter ablation for ventricular tachycardia among elderly patients
Source: J Interv Card Electrophysiol. 2026 Apr 1;69(6):1431–43. doi: 10.1007/s10840-026-02316-8 (PMC13646012; doi:10.1007/s10840-026-02316-8)
Supplement: Supplementary file 1 [file 10840_2026_2316_MOESM1_ESM.docx]

**Supplemental material:**

**Table S1. Procedural complications**

| Age / Gender | Etiology | Description |
| --- | --- | --- |
| 74 / Male | NICM | Femoral artery pseudoaneurysm successfully managed with manual compression  In-hospital death due to ventricular arrhythmia |
| 77 / Female | NICM | Femoral artery bleeding required blood transfusion |
| 82 / Male | NICM | In hospital death due to ventricular arrhythmia |
| 82 / Male | DICM | Pulseless ventricular tachycardia at a recovery room needed resuscitation and emergent repeated ablation |
| 87 / Male | DCM | In-hospital death due to ventricular arrhythmia |
| 71 / Male | ICM | In-hospital death due to heart failure |
| 72 / Male | ICM | In-hospital death due to cardiogenic shock |
| 73 / Male | ICM | In-hospital death due to VA |
| 73 / Male | ICM | In-hospital death due to VA |
| 74 / Male | ICM | In-hospital death due to heart failure |
| 74 / Male | ICM | Retroperitoneal hemorrhage requiring blood transfusion |
| 75 /Female | ICM | In-hospital death due to ventricular arrhythmia and heart failure |
| 76 / Male | ICM | Limb ischemia due to ECMO cannula requiring  In-hospital death due to heart failure |
| 76 / Female | ICM | Pericardial effusion requiring pericardiocentesis |
| 85 / Male | ICM | In-hospital death due to VA |

*Abbreviations: ECMO, extracorporeal membrane oxygenation; ICM, ischemic cardiomyopathy; NICM, non-ischemic cardiomyopathy; VA, ventricular arrhythmia.*
